# A Quantitative PCR-Electrochemical Genosensor Test for the Screening of Biotech Crops

**DOI:** 10.3390/s17040881

**Published:** 2017-04-18

**Authors:** Suely Moura-Melo, Rebeca Miranda-Castro, Noemí de-los-Santos-Álvarez, Arturo J. Miranda-Ordieres, José Ribeiro dos Santos Junior, Rosana A. da Silva Fonseca, María Jesús Lobo-Castañón

**Affiliations:** 1 Departamento de Química Física y Analítica, Universidad de Oviedo, 33006 Oviedo, Spain; suelymouramelo@yahoo.com.br (S.M.-M.); mirandarebeca@uniovi.es (R.M.-C.); santosnoemi@uniovi.es (N.d.-l.-S.-Á.); amir@uniovi.es (A.J.M.-O.); 2 Departamento de Química, Centro de Ciências da Natureza, Universidade Federal do Piauí, Teresina 64049-550PI, Brazil; jribeiro@ufpi.edu.br; 3 Instituto de Ciencias Biológicas, Universidade de Pernambuco, Recife 50670-901PE, Brazil; rosanafonsecabr@gmail.com

**Keywords:** electrochemical genosensor, genetically modified crops, PCR, screening GMOs

## Abstract

The design of screening methods for the detection of genetically modified organisms (GMOs) in food would improve the efficiency in their control. We report here a PCR amplification method combined with a sequence-specific electrochemical genosensor for the quantification of a DNA sequence characteristic of the 35S promoter derived from the cauliflower mosaic virus (CaMV). Specifically, we employ a genosensor constructed by chemisorption of a thiolated capture probe and *p*-aminothiophenol gold surfaces to entrap on the sensing layer the unpurified PCR amplicons, together with a signaling probe labeled with fluorescein. The proposed test allows for the determination of a transgene copy number in both hemizygous (maize MON810 trait) and homozygous (soybean GTS40-3-2) transformed plants, and exhibits a limit of quantification of at least 0.25% for both kinds of GMO lines.

## 1. Introduction

Since the mid-1990s, the traditional gene pool available to the plant breeders has been significantly widened by genetic engineering, giving rise to a large number of transgenic cultivars [1]. The ability of selecting and extracting the gene of interest from one organism and inserting it into the host plant genome to obtain the desired characteristic or trait has revolutionized agricultural practices over the last years. However, the increasing cultivation of genetically modified crops has been matched by a strong scientific and social debate still open [2]. In this regard, the fast pace of modern life and global markets seems to be incompatible with the time required to clarify remaining uncertainties as those related to long-term effects on human and animal health, as well as on global ecology.

In the meantime, most governments have recognized citizens’ full right to know food ingredients to protect themselves from possible harmful effects and to choose what to consume. In the European Union (EU), the presence of genetically modified (GM) material in food and feed must be labeled, unless its presence is considered accidental or technically unavoidable. As a result, threshold levels have been set at 0.9% and 0.5% for authorized and non-authorized genetically modified organisms (GMOs), respectively [3,4]. Verification of compliance with EU GMO legislation therefore entails detecting GMOs, assessing the legality of the constructs inserted, and determining the need for labeling. Nevertheless, the increasing number of genetically modified plant species, the diversity of incorporated genetic elements and the lack of global harmonized regulations [5,6] fuel the complexity of this task.

To tackle this challenge in an economically viable manner and within a reasonable timeframe, an initial screening step is desirable to reveal if the tested material is positive or negative in GMOs and then to proceed with more specific methods, if appropriate. Since modifications leading to new traits in plants are performed at DNA level and the integrity of the new encoded proteins is compromised in processed products, analysis at DNA level rather than at the gene product level is preferred. Undoubtedly, the mandatory inclusion of a single sequence not present in natural DNA sources, playing the role of a universal GMO molecular label, would make the detection of all transgenes much easier. The reality is unfortunately different, and a plethora of DNA-based screening methods, targeting the most frequently used elements in transgenic constructs inserted into food plants, has been developed [7]. After a statistical analysis, Cauliflower Mosaic Virus 35S promoter, CaMV35S or P35S, has turned out to be the predominant regulatory element [8].

Apart from polymerase chain reaction (PCR) and its fluorescent real-time variant (qPCR), both universally accepted GMO detection methods, new technologies are emerging whose efforts are aimed at fulfilling the desired characteristics for GMO screening, i.e., simplicity, rapidity, low cost, specificity and suitability for in-field analysis. Among them, electrochemical genosensors occupy a prominent place [9,10].

Electrochemical genosensors are a subclass of chemical sensors in which the formation of Watson-Crick base pairs between a single-stranded DNA probe, attached to an electrochemical transducer (the immobilized recognition element), and a complementary DNA target produces an electrochemical signal that is related to the concentration of the analyte being studied.

To date, genosensors have demonstrated their ability to detect small DNA or RNA oligonucleotides quickly and sensitively; they have also been employed in the agro-food field [11,12]. However, their applicability beyond synthetic DNA fragments has been limited by genomic DNA complexity, particularly problematic in plants due to their rampant polyploidy. Since it is intended to detect a small single-stranded DNA sequence within a complete genome, that is, large double-stranded DNA with strong internal secondary structure, this is by no means a minor issue. Consequently, it is usually required to combine the genosensor with a previous nucleic acid amplification process that allows the restriction of the target DNA size, thus facilitating its surface hybridization, while improving method sensitivity.

In this work, we propose the combination of a highly selective electrochemical genosensor with a previous PCR-amplification step for the screening of biotech crops by using a DNA sequence specific to the promoter CaMV35S. The methodology has been validated using certified reference materials of MON810 maize and GTS40-3-2 soybean, which are hemizygous and homozygous for the transgene, respectively.

## 2. Materials and Methods

### 2.1. Instrumentation

**Figure 1:**
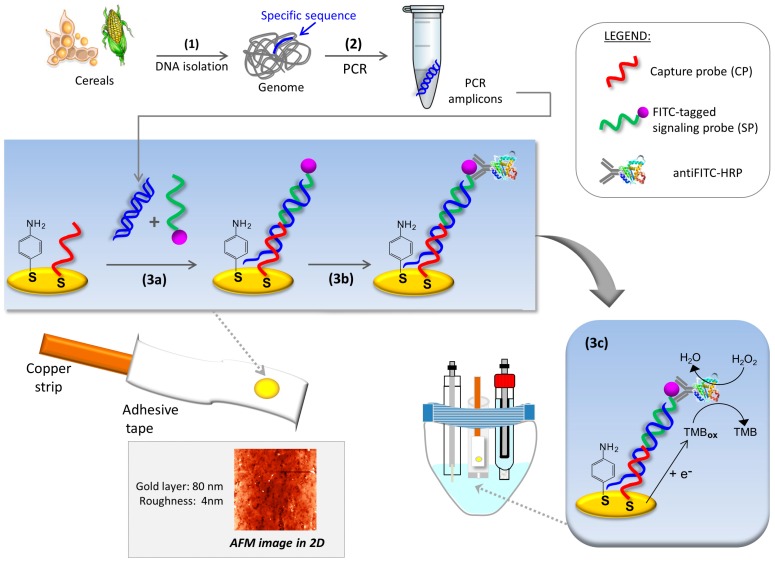
Schematic representation of the PCR-electrochemical genosensor method: (**1**) sample preparation involving DNA isolation; (**2**) PCR amplification of P35S; (**3a**) entrapment of PCR amplicons on the sensing layer using a fluorescein isothiocyanate (FITC)-tagged signaling probe; (**3b**) enzyme labeling with anti-FITC-horseradish peroxidase (HRP) conjugate; (**3c**) chronoamperometric detection of the oxidized tetramethylbenzidine (TMB) enzymatically generated. Inset: AFM micrograph of gold surfaces obtained by physical vapor deposition.

Electrochemical measurements were performed with a conventional three-electrode electrochemical cell driven by a computer-controlled μAutoLab type II potentiostat with GPES 4.9007 software (EcoChemie B.V., Utrecht, The Netherlands). All of the potentials are referred to the Ag|AgCl|KCl(sat)|KNO_3_(sat) reference electrode. A platinum wire and a 2-mm diameter homemade gold electrode acted as auxiliary and working electrodes, respectively. The working electrode was obtained by physical vapor deposition of gold wire (99.9% purity) in high vacuum on a polyester workpiece previously treated at 365 nm for 5 days. Atomic Force Microscopy (AFM) micrographs of the resulting thin gold films showed an averaged thickness of 80 nm with a roughness of 4 nm (Figure 1, Inset). A copper strip was used for electrical contact and the electrochemical area was defined by an adhesive tape with a circular hole of 0.0314 cm^2^. Chronoamperometric measurements were performed by immersing the homemade working electrode in a 500-µL microcell designed by our group.

### 2.2. Reagents

Synthetic oligonucleotides were purchased as lyophilized desalted powder from Integrated DNA Technologies (IDT, Leuven, Belgium). Their sequences are shown in Table 1. The commercially supplied disulfide-modified capture probes were treated with dithiothreitol (DTT) and then purified by elution through a Sephadex G25 column (NAP-10, GE Healthcare, Madrid, Spain) to yield the respective thiolated oligonucleotides as described elsewhere [13]. All oligonucleotide stock solutions were aliquoted and diluted to convenient concentrations (GENESYS^TM^ 10S UV-Vis Spectrophotometer, Thermo Scientific, Madrid, Spain) and stored at −20 °C. *p*-Aminothiophenol (*p*-ATP), bovine serum albumin (BSA), DTT, 3,3′,5,5′-tetramethylbenzidine (TMB) in a ready-to-use format (K-blue enhanced activity substrate, including H_2_O_2_), concentrated saline sodium phosphate-EDTA (20× SSPE) pH 7.4 and phosphate buffer saline (10× PBS) were all purchased from Sigma-Aldrich (Madrid, Spain) and used without further purification. 1% casein blocking solution in PBS was acquired from Thermo Scientific. Fab fragments from polyclonal anti-fluorescein antibodies conjugated to horseradish peroxidase (anti-FITC-HRP) were purchased from Roche Diagnostics GmbH (Mannheim, Germany). DNA intercalating dye EvaGreen^®^ (20×, Biotium, Fremont, CA, USA) and ROX reference dye (50×, Invitrogen, Madrid, Spain) were used for real-time fluorescence-based PCR monitoring. All other reagents were of analytical grade and used as received. Unless otherwise stated, aqueous solutions were prepared using high purity deionized water (18 MΩ·cm resistivity) produced with a Milli-Q System (Direct-Q^®^, Millipore Corporation, Madrid, Spain).

### 2.3. Sample Preparation and DNA Extraction

Certified reference materials in the form of homogenized seed powder of GM maize event MON810 [0, 2 and 10% (*w*/*w*)] and GM soy event GTS40-3-2 [10% (*w*/*w*)] were obtained from the Institute of Reference Materials and Measurements (IRMM), Joint Research Centre (JRC), European Commission (EC), through Sigma-Aldrich, Spain. Soybean flour from ecological agriculture was purchased from a local eco-market (Oviedo, Spain).

Genomic DNA was extracted and purified from 100 mg of homogenized flour using the Nucleospin^®^ food kit (Macherey-Nagel, Düren, Germany), according to the manufacturer’s instructions with minor modifications as described in Reference [14]. All extractions included a blank for controlling potential contamination during the procedure. UV spectrophotometry (Cary 60 Agilent including a mirrored ultramicrovolume cuvette cap) was used to evaluate the purity of extracts (A_260_/A_280_ ratio around 1.8) and to determine DNA concentration. To ensure the quality of the extracted DNA for PCR amplification, the chloroplast rbcL gene was amplified as a universal control for plants [15]. DNA extracts (100 ng/µL) were aliquoted and stored at −20 °C until use, minimizing repetitive freeze-thaw cycles to protect sample integrity.

### 2.4. Real-Time Polymerase Chain Reaction (qPCR)

Real-time PCR reactions were conducted in 25 µL of PCR mixture containing 50 ng of total DNA (2 µL), 1× buffer PCR, 2.5 mM MgCl_2_, 250 µM dNTPs, 0.2 µM each P35S primer (P35S-FP and P35S-RP), 1.25 U Immolase^TM^ DNA polymerase (Ecogen, Madrid, Spain), and including the DNA intercalating dye 0.2× EvaGreen^®^ and 1× ROX reference dye. Amplifications were run in the ABI Prism^®^ 7900HT Sequence Detection Instrument (Applied Biosystems, Madrid, Spain) programmed as described below.

Initial denaturation was set for 10 min at 95 °C to activate the polymerase, after which 50 cycles of amplification were performed with denaturation at 95 °C for 30 s, followed by annealing at primer-specific temperature and elongation at 65 °C for 1 min. Fluorescence was measured after each cycle and data analysis was performed with the software SDS version 2 (Applied Biosystems). After the cycling program, melting curve analysis was performed by cooling to 60 °C for 2 min and then increasing the temperature to 95 °C with a slope of 1.75 °C/s while measuring the fluorescence continuously.

### 2.5. End-Point Polymerase Chain Reaction (PCR)

The end-point PCR amplifications were performed with the same mix composition as described for real-time PCR but removing the fluorescent dyes. Reactions were run in a thermal cycler (GeneAmp^®^ PCR System 2700 thermocycler (Applied Biosystems)) under the following conditions: 10 min at 95 °C (hot-start/denaturation), followed by 38 repetitions of 30 s at 95 °C (denaturation), and 60 s at 65 °C (annealing and extension). Each sample, including all controls, was analyzed in triplicate.

### 2.6. Agarose Gel Electrophoresis

Electrophoresis was conducted in 1× TBE buffer (89 mM Tris-borate, and 2 mM EDTA, pH 8.3) on a 3% (*w*/*v*) agarose gel matrix, stained with ethidium bromide (0.5 µg/mL). Separation was performed at 80 V for ~60 min. DNA bands were visualized by UV light and their size was compared to a known PCR Low Ladder Marker Set (20–1000 bp, Invitrogen) to determine the approximate number of base pairs of the amplicon.

### 2.7. Electrochemical Genosensor

The genosensors were constructed by dropping on the gold electrodes 6 µL of a 0.1 µM solution of the thiolated capture probe (CP) in 2× SSPE, pH 7.4, and incubating overnight at 4 °C. The DNA-modified surfaces thus obtained were further incubated with 6 µL of 1 mM *p*-aminothiophenol in 2× SSPE, pH 7.4 for 50 min [14]. The genosensors were used in a sandwich-type hybridization assay, schematically depicted in Figure 1. First, 10 µL of the PCR-amplified sample without any previous purification was added to 90 µL of the hybridization buffer solution (2× SSPE, pH 7.4), containing 2.5% BSA and 0.1 µM FITC signaling probe. A great excess of FITC signaling probe and BSA were used to assist in the homogeneous hybridization reaction, circumventing typical thermal denaturation. After incubation at room temperature in darkness for 30 min, the working surface was covered with 6 µL of the abovementioned solution for 2 h. Upon washing with blocking buffer (1% casein (*w*/*v*) in 1× PBS, pH 7.4) to prevent nonspecific adsorption of the enzyme conjugate, anti-FITC-HRP, the modified surface was incubated with 0.5 U enzyme/mL in blocking buffer for 30 min at room temperature and protected from light. Finally, after washing with 1× PBS pH 7.4, the working electrode was immersed into 450 µL of commercial TMB solution and, after 30 s of incubation, the enzymatically oxidized TMB was reduced at 0 V, recording the current during 1 min.

## 3. Results and Discussion

A schematic representation of the proposed method for controlling the presence/absence of genetically modified organisms in food and feed is depicted in Figure 1. After DNA extraction, specific PCR amplification of Cauliflower Mosaic Virus 35S promoter (GenBank accession number AY326434) was performed. Then, enzyme-amplified electrochemical detection of the amplicons was carried out. The chronoamperometric detection of the specific DNA fragment of P35S was based on a sandwich hybridization assay previously optimized by our group. In short, the genosensor was prepared on a gold thin-film previously washed with ethanol and water, and dried under a stream of nitrogen gas. After conditioning, a binary self-assembled monolayer composed of a thiolated capture probe (CP) and *p*-aminothiophenol (*p*-ATP) was built following a backfilling strategy described elsewhere [14]. This thioaromatic agent leads to lower background signals in comparison to the typically used 6-mercaptohexanol, and improvement in sensing surface stability [16]. Hybridization experiments were performed in a sandwich-like format to maximize the selectivity of the assay, see Materials and Methods.

### 3.1. PCR Amplification: Design and Evaluation

The combination of a sandwich-format genosensor with a previous PCR DNA amplification entails some extra cautions in primer design to ensure reliable results. Although the target sequence can include the PCR primers, it is highly advisable to avoid primers hybridization and/or competition for the target sequence with capture and signaling DNA probes. As a result, the target sequence is located at the center of the amplicon and hanging single-stranded DNA segments will be present in the surface-confined ternary duplex, instead of the perfect duplex traditionally sought when a genosensor is designed. A pair of primers, previously designed for isothermal amplification of a P35S specific sequence, was here re-examined for exponential heterothermic DNA amplification. Despite leading to a 21-nucleotide protruding tail adjacent to the transducer surface, no detrimental effect on surface hybridization efficiency was observed [14].

**Figure 2:**
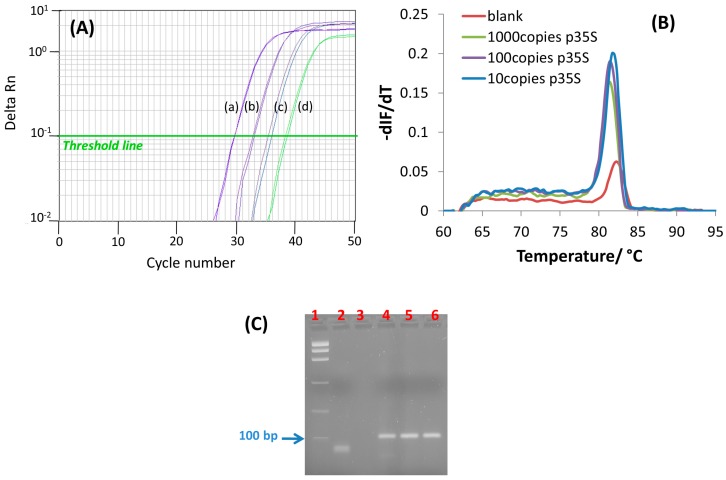
(**A**) Amplification curves obtained for real-time fluorescence-based PCR of genomic DNA extracted from MON810 maize flour. The initial number of P35S copies per assay was (**a**) 10^3^, (**b**) 10^2^, (**c**) 10, (**d**) non-template control. The horizontal green line indicates the threshold level used to establish the calibration curve; (**B**) Fluorescence-based melt curves recorded after the real-time PCR amplification experiments presented in (**A**); (**C**) 3% Agarose gel electrophoresis (in 1× TBE buffer) of PCR products from different initial number of P35S copies per assay (lane 1, ladder; lane 2, non-template control, lane 4, 10 copies, lane 5, 10^2^ copies; lane 6, 10^3^ copies).

The application of a fine-tuned primer set for isothermal DNA amplification, helicase-dependent amplification (HDA) or recombinase polymerase amplification (RPA), to PCR has reasonable chance of succeeding [17]. Instead, the opposite situation in which primers designed for PCR are used for amplification at a constant temperature, although possible, could be more demanding. In this work, we designed a PCR thermal profile by simply including a 95 °C denaturation step before the annealing-extension step at a primer-specific temperature (65 °C), see Materials and Methods. The theoretical two-fold increase in target sequence after each PCR cycle was studied by real-time fluorescence, using EvaGreen^®^ as double-stranded DNA-binding fluorescent dye, and the corresponding signal was normalized against the internal reference dye signal (ROX signal). Figure 2 shows the PCR amplification curves recorded from serial dilutions (10-fold) of genomic DNA isolated from 10% (*w*/*w*) MON810 maize GM-flour. An exponential enhancement of fluorescence as a function of the number of PCR cycles was found in accordance with an increasing amount of amplified ds-DNA product (Figure 2A). The number of PCR cycles at which the amount of amplified product crosses the threshold limit and generates a detectable fluorescence signal above the noise (green line in Figure 2A) was reduced as the starting template amount increased. Likewise, a linear dependence between the threshold cycle (C_T_) and the logarithm of the initial number of DNA template copies was found:

C_T_ = (−3.2 ± 0.2) log_10_ (DNA copy number) + (39 ± 1); r = 0.994

The calculation of the target copy number (P35S copies) took into consideration an estimated 2.6 pg haploid genome weight (C-value) for maize as reported in [18] and the hemizygous contribution of the MON810 construct to the IRMM certified standard.

From these analyses, PCR efficiency (E) can also be calculated according to the equation:

E = (10^−1/slope^) − 1

and expressed in percentage (%). A value of 104% was obtained, which is in agreement with the European Network of GMO Laboratories (ENGL) acceptance criteria for q-PCR (slope between −3.6 and −3.1, corresponding to amplification efficiencies of 90 and 110%, respectively) [19].

To clarify the specificity of the real-time fluorescence signals, melting curve analysis was performed immediately after PCR amplification (Figure 2B). The presence of only one melting peak at 82 °C was in line with the presence of only one PCR product in the samples. However, the ds-DNA product generated after 38 PCR cycles in the absence of a DNA template (curve d, Figure 2A) led to a less intense peak, but of the same melting temperature (T_m_). On the other hand, electrophoretic analysis of the PCR-amplified GM-maize genome showed bands of the same length in all samples, corresponding to the 105-bp amplified region (Figure 2C). Nevertheless, unlike the results from melting curve analysis, no band corresponding to the amplified specific sequence was observed in the non-template control; only a band of lower length ascribable to the primers was apparent. The discrepancy between both detection methods could be due to their differences in sensitivity, as the affinity of ethidium bromide towards ds-DNA is significantly lower than that for EvaGreen^®^. An aerosol contamination from the MON810 sample could also be a reasonable explanation of the weak signal recorded for the non-template control. Whatever the reason for spurious amplification is, the combination of closed-tube PCR amplification with a sequence-specific detection method, i.e., an electrochemical genosensor, would help to circumvent erroneous results.

### 3.2. Combining PCR and Electrochemical Genosensor

Due to its exponential nature, PCR is an extremely powerful DNA amplification tool. However, in order to take advantage of all this potential for quantitative analysis, long reaction times are discouraged; as depletion of reaction components and/or polymerase decline can occur, with the concomitant leveling-off in amplicon concentration. Trying to maintain a relationship between the initial transgenic DNA amount and the recorded electrochemical response, the number of PCR thermal cycles was adjusted on the basis of the previous real-time PCR results. Since positive amplification for the non-template control was observed after 38 cycles, this value was chosen to carry out the ended-point PCR in the absence of fluorescent dyes. For this purpose, different dilutions of the transgene were obtained by mixing different quantities of maize DNA extracted from 10% (*w*/*w*) MON810 GM flour with non-GM maize DNA to a final amount of ~50 ng, and at least three replicates of each diluted GM maize DNA were amplified. The generated P35S copies from MON810 maize were challenged to the electrochemical genosensor without any previous purification, but they were diluted 10 times with the hybridization buffer containing 0.1 µM FITC-signaling probe in order to perform the homogeneous hybridization step.

**Figure 3:**
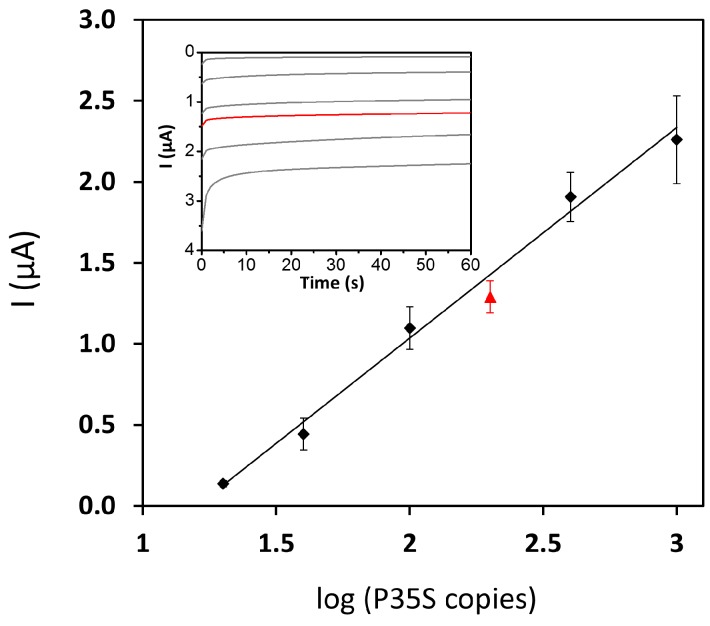
Analytical response in absolute value of the PCR-electrochemical genosensor method for MON810 maize: (black dots) calibration plot obtained by dilution of certified MON810-10% with MON810-0%; (red triangle) reference material MON810-2%. Inset: chronoamperograms corresponding to the data displayed in the calibration plot.

Figure 3 shows the cathodic currents recorded chronoamperometrically as a function of the initial P35S copies in MON810 GM-maize. A linear calibration [I (µA) = −1.3 (±0.1) log_10_ (P35S copies) + 1.6 (±0.2); r = 0.997] was accomplished from 20 to 1000 copies, that is, from 0.2% to 10% of MON810 GM maize. It is important to point out that 0% MON810 certified reference material gave a similar response to that from the ordinary blank of the electrochemical genosensor, suggesting no interferences between “background” genomic DNA and PCR reagents with the designed genosensor. A limit of quantification (LOQ) of 20 P35S copies (0.2% MON810) was estimated as the lower value of the linear calibration. Relative standard deviation (RSD) values were in the range of 8 to 22%, with an average value of 14%. Furthermore, with the aim of determining the reliability of this calibration plot, we performed the complete assay for 2% (*w*/*w*) MON810, starting from the commercially available certified standard instead of obtaining it from the dilution of MON810-10% with MON810-0%. The current intensity corresponding to the reference material 2% (*w*/*w*) MON810 was −1.3 (±0.1) µA (Figure 3). When this value is introduced in the previous calibration equation, a P35S copy number of 157 ± 38 was obtained in good agreement with the ~192 copies of P35S expected in 50 ng of MON810-2%.

Finally, given that promoter CaMV 35S is a common regulatory element in transgenic crops, the proposed methodology was used to test the GMO content in flour soybean standards with a certified percent in GTS40-3-2, also known as Roundup-Ready^®^ Soybean (RRS), which possesses a homozygous character when inserted into the soy genome (C-value for soybean: 1.13 pg [18]). The procedure was equivalent to that carried out with MON810 GM-maize. The purchased 10% (*w*/*w*) RRS standard was diluted, after DNA isolation, with the 0% (*w*/*w*) RRS and subsequently subjected to 38 PCR cycles before analysis with the electrochemical genosensor.

**Figure 4:**
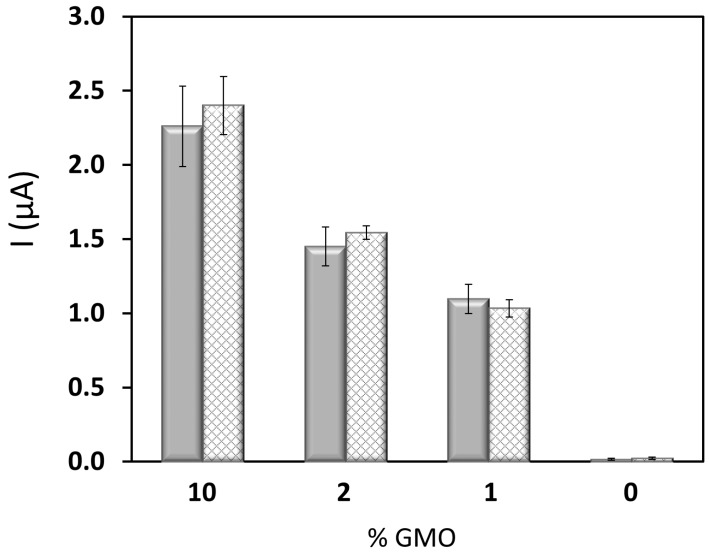
Variation of the chronoamperometric signal, in absolute value, recorded using the PCR-electrochemical genosensor method with GMO percentage. MON810 maize (solid grey), GTS40-3-2 (striped grey).

Again, a linear dependence of the analytical signal with the logarithm of the starting P35S copies in RRS was obtained [I (µA) = −1.23 (±0.06) log_10_ (P35S copies) + 2.1 (±0.2); r = 0.996], between 100 and 4000 copies, that is, between 0.25% and 10%. In this case, an LOQ of 100 P35S copies, equivalent to 0.25% RRS, was estimated as well as an average RSD of 8%. Strikingly, the slope of this semilogarithmic calibration perfectly matched up that for the calibration curve built with certified MON810 standards. In order to determine the generality of our approach we plotted the genosensor response as a function of the % GMO in the assay (Figure 4). We found similar results for the two plant lines. These findings support the possibility of using a single calibration to detect and quantify the promoter P35S in all GM crops containing this promoter. However, it is important to note that in this particular case the homozygous plant, which contains twice as many copies of the transgene as the hemizygous one, has about half the C-values. Therefore, the single calibration must be excluded, as is the case in real-time PCR measurements [20].

The proposed method combining PCR with an electrochemical genosensor for the detection of the regulatory element CaMV35S has analytical performance that allows its use for the control of the compliance with the current EU legislation on GMOs (the LOQ is clearly below the threshold value established by law). Furthermore, unlike our prior reported work that couples this electrochemical hybridization assay with a helicase-dependent isothermal DNA amplification [14], this methodology allows not only Yes/No detection of GMOs but also quantification of the 35S promoter, widely used in the constructs inserted in GM crops.

## 4. Conclusions

Abundant literature has been published in recent years reporting different genosensors designed for GMO analysis, mainly targeting the promoter P35S, one of the most frequently used elements in transgenic constructs inserted into food plants. As a proof of concept, they have demonstrated their potential with short synthetic oligonucleotides, but most of them do not step into the real world of analyzing genomes. Few cases have been challenged with food samples but, considering the performance (limit of detection and sensitivity) obtained with short oligo targets, their successful results in complex samples become hard to explain [21,22]. Commercial samples have been recently evaluated, finding, after extensive enzymatic digestion and no amplification, they were non-transgenic products [23]. Nevertheless, without verifying the quality of DNA after such a destructive treatment, this conclusion turns out to be unsupported. Likewise, the addition of known amounts of short synthetic ssDNA to blank samples is far from a real sample evaluation.

In contrast to most methods reported, here we faced the detection of GMO using positive real samples. The combination of a PCR amplification step and an electrochemical sequence-specific sensor has been proven to be an attractive method for the quantification of the promoter P35S. The assay allows the establishment of a correlation between the electrochemical response and the initial amount of DNA to quantify the GMO% in flour samples, unlike most methods that provide calibration in nM DNA units, which is meaningless for regulatory purposes. The absolute limit of quantification was estimated below 100 copies of the transgene for both hemizygous and homozygous genetically modified plants, corresponding to less than 0.25% of GMO. This quantitative P35S test can thus be used for the efficient screening of potential GMO presence in food and agriculture products.

## Figures and Tables

**Table 1:** Probes and primers sequences used in this work.

Sequence Name	Length	Oligonucleotide Sequences 5′ → 3′
Capture probe (CP)	27 nt	AGA GGA AGG GTC TTG CGA AGG ATA GTG-(CH_2_)_6_SH
Signaling probe (SP)	53 nt	^1^FITC-CTA GAG TCA GCT TGT CAG CGT GTC CTC TCC AAA TGA AAT GAA CTT CCT TAT AT
P35S-Forward primer (P35S-FP)	27 nt	GTA AGG GAT GAC GCA CAA TCC CAC TAT
P35S-Reverse primer (P35S-RP)	27 nt	TCT GCT AGA GTC AGC TTG TCA GCG TGT
Plant-Forward primer (Plant-FP)	23 nt	CTT GAT TTT ACC AAA GAT GAT GA
Plant-Reverse primer (Plant-RP)	20 nt	TTC TTC GCA TGT ACC CGC AG

^1^ FITC Fluorescein isothiocyanate.
